# Immunostimulatory Activities of CpG-Oligodeoxynucleotides in Teleosts: Toll-Like Receptors 9 and 21

**DOI:** 10.3389/fimmu.2019.00179

**Published:** 2019-02-08

**Authors:** Chao-Yang Lai, Guann-Yi Yu, Yunping Luo, Rong Xiang, Tsung-Hsien Chuang

**Affiliations:** ^1^Immunology Research Center, National Health Research Institutes, Zhunan, Taiwan; ^2^National Institute of Infectious Diseases and Vaccinology, National Health Research Institutes, Zhunan, Taiwan; ^3^Deptartment of Immunology, Chinese Academy of Medical Science, School of Basic Medicine, Peking Union Medical College, Institute of Basic Medical Science, Beijing, China; ^4^Collaborative Innovation Center for Biotherapy, School of Basic Medical Science, Chinese Academy of Medical Science and Peking Union Medical College, Beijing, China; ^5^Department of Immunology, School of Medicine, Nankai University, Tianjin, China; ^6^International Joint Center for Biomedical Research of the Ministry of Education, Tianjin, China; ^7^Program in Environmental and Occupational Medicine, Kaohsiung Medical University, Kaohsiung, Taiwan

**Keywords:** adjuvant, CpG-ODN, immune modulator, innate immunity, toll-like receptor

## Abstract

Toll-like receptors (TLRs) are pattern-recognition receptors that detect a wide variety of microbial pathogens for the initiation of host defense immunological responses. Thirteen TLRs have been identified in mammals, and teleosts contain 22 mammalian or non-mammalian TLRs. Of these, TLR9 and TLR21 are the cytosine-phosphate-guanosine-oligodeoxynucleotides (CpG-ODNs) recognition TLRs in teleosts. TLR9 is a mammalian TLR expressed in teleost but not in the avian species. TLR21 is a non-mammalian TLR expressed in both teleost and the avian species. Synthetic CpG-ODNs are potent immunostimulants that are being studied for their application against tumors, allergies, and infectious diseases, and as a vaccine adjuvant in humans. The immunostimulatory effects of CpG-ODNs as vaccine adjuvants and their antimicrobial function in domestic animals and teleosts are also being investigated. Most of our current knowledge about the molecular basis for the immunostimulatory activity of CpG-ODNs comes from earlier studies of the interaction between CpG-ODN and TLR9. More recent studies indicate that in addition to TLR9, TLR21 is another receptor for CpG-ODN recognition in teleosts to initiate immune responses. Whether these two receptors have differential functions in mediating the immunostimulatory activity of CpG-ODN in teleost has not been well-studied. Nevertheless, the existence of two recognition TLRs suggests that the molecular basis for the immunostimulatory activity of CpG-ODN in teleosts is different and more complex than in mammals. This article reviews the current knowledge of TLR9 and TLR21 activation by CpG-ODNs. The key points that need to be considered for CpG-ODNs as immunostimulants with maximum effectiveness in activation of immune responses in teleosts are discussed. This includes the structure/activity relationship of CpG-ODN activities for TLR9 and TLR21, the structure/functional relationship of these two TLRs, and differential expression levels and tissue distributions for these two TLRs.

## Introduction

Toll was originally identified in *Drosophila* as a type I transmembrane receptor involved in embryo development, and it plays an important role in innate immune responses to microbial infection in the adult fly (1–3). Thirteen toll-like receptors (TLRs), TLR1 to TLR13 were subsequently identified across all mammalian species, and humans contain ten of them, TLR1 to TLR10 (4–12). Human TLRs are well-investigated. These receptors can be divided into three subfamilies and play an essential role in innate immunity by recognizing a wide variety of pathogen-associated molecular patterns (PAMPs) from microbes (9–12). Phylogenetically, TLR1, TLR2, TLR6, and TLR10 are most closely related. TLR2 recognizes a broad range of microbial components, including lipoproteins, peptidoglycan, lipoteichoic acids, lipoarabinomannan, and zymosan (13–19). TLR2 and TLR6 form a complex that is more specific to triacyl lipopeptides; whereas, a heterodimer composed of TLR2 and TLR1 selectively recognizes triacyl lipopeptides (20–22). Ligand recognition of TLR10 has not been well-investigated; however, a recent paper showed that this TLR is a receptor for double-stranded RNA (dsRNA) (23). TLR4 is closely related to TLR5, with the former being responsible for recognizing lipopolysaccharides on the outer membrane of gram-negative bacteria and the latter recognizing flagellin, which is a component of bacterial flagella (24, 25). TLR3, TLR7, TLR8, and TLR9 comprise a TLR subfamily. These TLRs recognize nucleic acid-derived microbial PAMPs. TLR3 is activated by dsRNA generated during viral replication in infected cells (26). TLR7 and TLR8 recognize single-stranded (ss)RNA from viruses (27, 28). TLR9 is a receptor for microbial unmethylated cytosine-phosphate-guanosine (CpG) DNA (29, 30).

TLRs contain an extracellular domain (ectodomain) comprising multiple leucine-rich repeats (LLRs), a cysteine-rich motif followed by a transmembrane region, and a highly conserved cytoplasmic toll/interleukin (IL)-1 receptor (TIR) domain. The TLR ectodomain is the location of ligand binding, while the cytoplasmic TIR domain provides a key site for intracellular signaling (31, 32). Upon activation by ligand ligation, TLR monomers become dimerized. Their cytosolic domains subsequently recruit adaptor proteins from the myeloid differentiation primary response 88 (MyD88) family. These include MyD88, TIR-domain-containing adapter-inducing interferon-β (TRIF)/TIR domain-containing adapter molecule 1 (TICAM1), TIR domain-containing adapter protein (TIRAP)/MyD88 adapter-like (Mal), toll/interleukin-1 receptor protein (TIRP)/toll-like receptor adaptor molecule (TRAM), and SRAM; thereby, initiating downstream signaling pathways (31). All TLRs, except for TLR3, signal via a MyD88-dependent pathway. TLR3 and TLR4 utilize a TRIF-dependent pathway for signaling. In the MyD88-dependent pathway, a MyD88/IL-1R-associated kinase 1 (IRAK1)/IRAK4/TNFR-associated factor 6 (TRAF6) complex activates transforming growth factor beta-activated kinase 1 (TAK1), which in turn promotes the activation of several transcription factors, including factor kappa-light-chain-enhancer of activated B cells (NF-κB) and activator protein 1 (AP-1). In the TRIF-dependent pathway, the TLR recruits TRIF to activate NF-κB, AP-1, and interferon response factors (IRFs). Activation of NF-κB and AP-1 is mediated by TRAF6 and receptor-interacting protein (RIP), and IRF3/7 activation involves a TBK1-IKKε/IKKi complex (33–35). These transcription factors are key regulators of the expression of adhesion and co-stimulatory molecules and the production of various inflammatory cytokines required for triggering of innate immune responses. This subsequently leads to the activation of adaptive immune responses (36–38).

The immunostimulatory properties of microbial DNA were first discovered in a DNA fraction of bacillus Calmette–Guerin (39, 40). Additional studies have revealed that the immune stimulatory activity is present only when the DNA contains unmethylated CpG deoxynucleotides (41, 42). Synthetic phosphorothioate-modified CpG-ODNs mimic the functions of microbial CpG-deoxynucleotides containing DNA (CpG-DNA). In mammals, CpG-ODNs induce a wide variety of immune responses. Antigen presentation is promoted in dendritic cells because of the increased antigen processing and upregulated expression of costimulatory molecules. Production of inflammatory cytokines from dendritic cells, monocytes, and macrophages are increased. B-lymphocytes are activated, resulting in an increased proliferation and immunoglobulin (Ig) secretion. Natural killer (NK) cells are activated to produce IFN-γ. T-lymphocytes are also affected, resulting in initiation of T-helper (Th)1 responses. Moreover, the generation of cytotoxic T lymphocytes is increased (43–45).

By *in vivo* studies with gene knockout mice and *in vitro* studies with cell-based TLR9 activation assay, TLR9 was identified to be the cellular receptor for CpG-ODN (29, 30, 46). In mammals, TLR9 is mainly expressed in dendritic cells, monocytes/macrophages, and B cells (47–49). Activation of TLR9 by CpG-ODN results in several immunological effects, including activation of dendritic cells, monocytes, macrophages, and NK cells leading to antigen presentation and the production of cytokines. In addition, induction of TLR9 activates B cells and increases B-cell proliferation. TLR9 activation upregulates Th1 polarized cytokine productions. Cytokines including TNF-α, IL-6, IL-12, interferons, and chemokines promote T cell activation. These immunologic responses resulted by TLR9 activation replicate the *in vivo* function of CpG-ODNs further confirmed that TLR9 is the major cellular receptor for CpG-ODN in mammals (45, 50).

Because of these immunostimulatory activities, CpG-ODNs are being investigated for their properties against tumors, allergies, and infectious diseases for humans (50). In the last quarter of 2017, CpG-ODN was approved for the first time for application in humans. Heplisav-B, a hepatitis B vaccine containing CpG-ODN as an adjuvant, was approved by the United States Food and Drug Administration. Two doses of the new vaccine were satisfactory for immunization compared with three doses of the current hepatitis B vaccines that contain aluminum hydroxide as an adjuvant (51, 52). In addition to their application in humans, CpG-ODNs are being investigated for their adjuvant and antimicrobial activities in other species, including domestic animals and teleosts (53–56). These studies reveal the potential usages of CpG-ODNs in human health, agriculture, and aquaculture.

## TLR9 and TLR21 Mediate the Immunostimulatory Activity of CPG-ODN

Other than the mammalian TLRs, several non-mammalian TLRs have also been identified in other vertebrate lineages (57, 58). For example, ten TLRs have been identified in avian genomes. Analysis of genomic DNA of two distantly related avian species, chicken and zebra finch, have identified: TLR1La, TLR1Lb, TLR2a, TLR2b, TLR3, TLR4, TLR5, TLR7, TLR15, and TLR21. The avian TLR1La, TLR1Lb, TLR2a, TLR2b, TLR3, TLR4, TLR5, and TLR7 are orthologs to the TLR found in mammals. The TLR1La and TLR1Lb result from duplication of TLR1-like genes, and TLR2a and TLR2b result from the duplication of TLR2 genes in avian evolution (59–63). Mammalian TLR7 and TLR8 have higher homology to each other than other TLRs, which could be due to a duplication of the same gene in some evolutionary duplication event (8). The avian genome contains TLR7 but does not contain TLR8. The mammalian TLR9 and TLR10 are also missing from the avian genomes. TLR15 and TLR21 found in the avian genome do not exist in genomes of mammalian species. TLR15 is phylogenetically related to the TLR2 family and appears to be unique to the avian species. In contrast, the avian TLR21 could be an ortholog to teleost and amphibian TLR21 (57–59). Interestingly, the avian species do not contain TLR9; however, like their actions in mammalian species, CpG-ODNs also activate marked immune responses and provide protection from microbial infections in chickens (50, 55, 64–67). Further studies have revealed that chicken TLR21 is a functional homolog to mammalian TLR9 in terms of responding to CpG-ODN stimulation (68, 69). The chicken TLR21 conferred cellular responses to CpG-ODN stimulation when it was over-expressed in human embryonic kidney (HEK) 293 cells. Knockdown of this receptor by shRNA significantly reduced the CpG-ODN-induced production of IL-1, IL-6, and iNOS from chicken DH11 cells (68, 69).

In teleosts, at least 22 different TLRs have been identified, including both mammalian (TLR1–TLR4, TLR5M, TLR5S, and TLR7–TLR9) and non-mammalian TLRs (TLR13, TLR14, TLR18–TLR28). In addition, orthologs of the mammalian signaling molecules and transcription factors for TLR functions have been identified (57, 58, 70–76). These TLRs are divided into six major subfamilies: TLR1, TLR3, TLR4, TLR5, TLR7, and TLR11 (57, 58).

The structure and ligand recognition properties of fish TLR1-3, 5, and 7–9 are similar to those of their mammalian counterparts. TLR2, a member of the TLR1 family, recognizes peptidoglycan, lipoteichoic acid, and lipopeptides. TLR3 detects dsRNAs. TLR5 recognizes bacterial flagellin. Teleost TLRs 7 and 8 respond to dsRNA, as well as to ssRNA, which is also recognized by mammalian TLR7 and TLR8 (57, 58). In contrast to mammalian TLR4, fish TLR4 does not recognize lipopolysaccharides (LPSs) despite its structural conservation with the former (77). Among non-mammalian TLRs, teleost TLR19 and TLR22 recognize dsRNAs (78–83). The recognition of dsRNAs by TLR19 results in the activation of IFN and NF-κB pathways and the protection of cells from infection by the grass carp reovirus (84). TLR22 recognizes dsRNAs to induce IFN production and protect cells from birnaviruses (83). In addition, a recent study showed that in fish, TLR22 functions as an equalizer for inflammation through the selective suppression of NF-κB and the activation of the MAPK pathway (85).

The immunologic effects of CpG-ODNs have been investigated in numerous teleost species. In these teleosts, much as in mammalian and avian species, CpG-ODNs upregulate the activation of macrophages, induce the proliferation of leukocytes, stimulate cytokine expression, and protect against bacterial, viral, and parasitic infections. Thus, CpG-ODNs have been studied for their application as antimicrobial agents and vaccine adjuvants in teleosts (53, 55). There is interest in the ligand recognition and functional properties of TLR9 and TLR21 in teleosts since these two TLRs have been shown to be the cellular receptors for CpG-ODN in mammals and chickens, respectively. TLR9 and TLR21 from zebrafish (*Danio rerio*) were comparatively investigated (86). Direct evidence to demonstrate that these two TLRs are the functional cellular receptors for CpG-ODN came from an experiment with cell-based activation assay in which the overexpression of both zebrafish (zeb)TLR9 and zebTLR21 in HEK293 cells conferred cellular responses to CpG-ODN stimulation (86). ZebTLR9 and zebTLR21 have different recognition profiles for CpG-ODNs with different nucleotide sequences. ZebTLR9 broadly recognizes CpG-ODN sequences that have higher activity for human cells and sequences that contain higher activity for mouse cells. In contrast, zebTLR21 prefers the CpG-ODNs that have higher activity for human cells (86). The biological functions of these two TLRs were investigated further in that study. CpG-ODNs that activate both zebTLR9 and zebTLR21 are more potent than others in the activation of cytokine productions in zebrafish and are more effective in protecting teleosts from the lethal effects of bacterial infection (86). These suggest that TLR9 and TLR21 cooperatively mediate the immunostimulatory effect of CpG-ODN in zebrafish. Beside these, the functions of TLR9 and TLR21 in other teleosts have not yet comparatively investigated.

## Structural Features for the Immunostimulatory Properties of CPG-ODN

Natural CpG-DNA in microbial genomes contains a phosphodiester backbone that is quickly degraded by nucleases *in vivo*. Thus, the phosphorothioate backbone was developed to create synthetic CpG-ODNs by replacing oxygen with sulfur in the phosphate group of the nucleic acid to make them more resistant to nucleases (87–89). Other than this, the immunostimulatory activity of CpG-ODN is also dependent on its nucleotide sequence and structure, and it may involve different strengths of activity in different species, known as “species-specific activity.” (90–92). Most of our knowledge about the structure-dependent activity and species-specific activity of CpG-ODNs come from studies of the interaction between CpG-ODNs and mammalian TLR9 (29, 30, 46, 90–92). Because previous studies used human and mouse cells which have TLR9 only, in addition the mammalian TLR9 was identified for investigation earlier than the non-mammalian TLR21 was.

Based on their structural features, CpG-ODNs are divided mainly into four classes. Class A (also known as type D) CpG-ODNs contain a central phosphodiester palindromic region with one or more CpG-motifs in the palindrome and consist of poly (G) sequences with a phosphorothioate backbone attached to the 5′ and 3′ ends. Class B (type K) CpG-ODNs contain a phosphorothiolate backbone throughout the entire sequence with several CpG-motifs. Class C CpG-ODNs contain phosphorothioate backbone with one or two CpG-motifs and a palindromic sequence at the 3′ end. The CpG-ODNs of class P contain two palindromic sequences with phosphodiester cytosines in the palindrome (90, 93–96). Table 1 shows the structures for the four classes of CpG-ODN. Different classes of CpG-ODNs have different immunostimulatory effects. Class A CpG-ODNs stimulate the production of large amounts of IFN-α and induce the maturation of plasmacytoid dendritic cells (pDCs) but have little effect on B-cell activation. Class B CpG-ODNs strongly induce B-cell proliferation, pDC and monocyte maturation, NK cell activation, and cytokine production. They also stimulate the production of IFN-α, but to a lesser extent than class A CpG-ODNs. The extent of the capability of class C CpG-ODNs to induce B-cell proliferation and IFN-α production is between that of class A and B CpG-ODNs. The immunological activities of class P CpG-ODNs are characterized by their high capability for inducing IFN-α production and NF-κB activation. Nearly all CpG-ODNs investigated in clinical trials have been class B CpG-ODNs (90, 93–96).

**Table 1:** Structural features of CpG-oligodeoxynucleotides (ODNs) in each of the four major classes.

**Class**	**Name**	**Sequence**
A	CpG-2336	5′- G*G*G*$\text{G}\underline{\text{-A-C-G-A-C-G-T-C-G-T-}}\text{G}$-G*G*G*G*G*G−3′
B	CpG-2007	5′- T*C*G*T*C*G*T*T*G*T*C*G*T*T*T*T*G*T*C*G*T*T-3′
C	CpG-2395	5′-T*C*G*T*C*G*T*T*T*T*C*G*G*C*G*C*G*C*G*C*C*G-3′
P	CpG-21798	5-$\underline{\text{T*C-}\text{G*T*C-G*A*C}\text{-G*A}}$*T*C-G*G*C*G*C-G*C*G*C*C*G-3

*Hyphens indicate phosphodiester and asterisks stand for phosphorothioate bonds. Red color shows CpG-hexamer and underlining indicates palindromic sequence*.

Another of the major structural features of CpG-ODNs is they include one or more copies of CpG-deoxynucleotide containing hexamer (CpG-hexamer) motifs. The immunostimulatory activity of these CpG-ODNs depends on the number, position, spacing, and surrounding bases of these CpG-hexamer motifs. Their species-specific activity is determined by the nucleotide context of these CpG-hexamer motifs (90–92). For example, CpG-1826, which contains two copies of the GACGTT-hexamer motif in 20 nucleotides, is more effective in activating murine cells than CpG-2007, which contains three copies of the GTCGTT-hexamer motif in 22 nucleotides; however, CpG-2007 is more potent in activating human cells than CpG-1826 (46, 90–92, 97). In addition, the nucleotide length of CpG-ODN plays a significant role in determining its immunostimulatory activity. In rabbit cells, CpG-C46 and CpG-C4609, which each contain 12 nucleotides and have a GACGTT- and AACGTT-hexamer motif, respectively, generate stronger immune responses than CpG-1826 and CpG-2007 (98).

## Sequence of CPG-ODN for TLR9 and TLR21 Activation in Teleosts

Several CpG-ODNs have been investigated in teleosts for their immunostimulatory activity and antimicrobial functions. There are well-written reviews for these properties of CpG-ODN in earlier works (53, 55). Table 2 summarizes the more recent work. Most of the CpG-ODNs used in these studies are class B. Like in mammals, CpG-ODN nucleotide length determines its immunostimulatory activity in teleosts. In Atlantic salmon (*Salmo salar*), CpG-ODNs that are 16–17 nucleotides long show less immunostimulatory effects that those that are 20–22 nucleotides long. CpG-ODNs shorter than 13 nucleotides lose their immunostimulatory properties. In addition, CpG-ODNs that are more than 30 nucleotides long have rarely been investigated for their immunologic activity in teleosts (53, 55, 120).

**Table 2:** Summary of CpG oligonucleotides used in teleost.

**CpG-ODN**	**Sequence (5^**′**^->3^**′**^)**	**Action**	**Fish**
2722	GTTGTCGTTTTTTGTCGTT	Induce NF-κB activation and cytokine expressions via TLR21 (99)	Grouper
2727	GTTGTCGTTTTTTGTGCTT	Induce NF-κB activation and cytokine expressions via TLR21 (99)	Grouper
1668	TCCATGACGTTCCTGATGCT	Activate innate and adaptive immune responses, and offer protection from bream iridovirus infection (100, 101) Used as an adjuvant for vaccines against V. harveyi infection (102) Activate innate immune response and upregulate TLR9 and IgM-mediated immune response (103) Increase protection against P. dicentrarchi infection (104) Stimulate upregulation of TLR9, IL-1and chemokine CC (105) Decrease sea lice infection in CpG-1668 fed group via induction of inflammatory gene expression (106, 107)	Rock bream Grouper Pacific red snapper Oliver flounder Cobia Atlantic salmon
2006	TCGTCGTTTTGTCGTTTTGTCGTT	Induce IgM and antimicrobial peptide gene expression (108) Stimulate IL-1 and IL-6 production and NF-κB activation in head kidney cells (109) Elicit better protection against E. tarda through activation of both TLR9 and TLR21 (86) Stimulate upregulation of IgM, TLR9, IL-1 and chemokine CC (105) Promote IgM secretion and upregulation of cd83, cd40, ifna1 and ifnb (110) Induce MAPK-activated protein kinase 2 activation in phagocytes (111)	Yellowtail Yellow croaker Zebrafish Cobia Atlantic salmon Atlantic salmon
2007	TCGTCGTTGTCGTTTTGTCGTT	Increase survival rates following challenge with E. tarda (112) Activate IL-1, IL-6 production and NF-κB activation in head kidney cells (109) Induce protective effect against S. iniae infection (113) Elicit better protection against E. tarda through activation of both TLR9 and TLR21 (86)	Olive flounder Yellow croaker Nile tilapia Zebrafish
2395	TCGTCGTTTTCGGCGCGCGCCG	Induce expression of antiviral Mx gene in spleen and liver (105) Upregulation of TLR21 expression (114)	Cobia Turbot
1013	CTCACTATCGTTCTTGATT	Increase WBC counts, peroxidase activity and oxidative radicals in head kidney, upregulate immune-related genes and enhance protection against S. iniae infection (115)	Asia sea bass
1670A	TCGAACGTTTTAACGTTTTAACGTT	Induce protective antiviral responses against grass carp reovirus (116)	Grass carp
1826	TCCATGACGTTCCTGACGTT	Activate IL-1, IL-6 production and NF-κB activation in head kidney cells (109)	Yellow croaker
C7	GGCGCGCGTCGCGCGCTA	Inhibite viral replication, promote proliferation of leukocytes, and enhance activation of head kidney phagocytes (117)	Olive flounder
205	GATCGCGTGCGTGCGTCTAT	Induce macrophage activation, leukocyte proliferation and protect against lethal E. tarda challenge (118)	Turbot
D	ACCGATAACGTTGCCAACGTTGGT	Upregulate leucocyte gene expressions including TNF-α, IL-1, TLR9, IRF-1, Mx, MHCIIa, IgM_H_ and CSF-1R (119)	Gilthead seabream

Compared to what is known about the critical role of CpG-hexamer motif in the activity of CpG-ODN in mammalian species, a conclusion has not been reached on what type of CpG-hexamer motif is best for generating a strong immune response in teleosts. CpG-1668, which contains one copy of the GACGTT-hexamer motif in 20 nucleotides, is reported to have immunostimulatory activity, adjuvant effects, and antimicrobial properties in different teleosts, including rock bream (*Oplegnathus fasciatus*), olive flounder (*Paralichthys olivaceus*), orange-spotted grouper (*Epinephelus coioides*), Asian sea bass (*Lates calcarifer*), and Pacific red snapper (*Lutjanus peru*) (100–104, 115). Moreover, when fed to Atlantic salmon (*Salmo salar*), CpG1668 induced the expression of cytokines, such as IL-1β and IL-12β, to protect this teleost fish from infection by sea lice (*Lepeophtheirus salmonis*), which are the most important ectoparasites that affect the farming of Atlantic salmon (106, 107). When administered to rock bream, CpG-1668 activates stronger protective effects against viral infection than other CpG-ODNs with GTCGTT-hexamer or with the same GACGTT-hexamer motif but with different nucleotide lengths (100). CpG-2006 and CpG2007, which contain three copies of the GTCGTT-hexamer motifs in 24 and 22 nucleotides, respectively, have been shown to induce immune responses in yellowtail (*Seriola quinqueradiata*), olive flounder, large yellow croaker (*Larimichthys crocea*), grass carp (*Ctenopharyngodon idella*), Nile tilapia (*Oreochromis niloticus*), and Atlantic salmon (108, 109, 112, 113, 116, 121). In olive flounder, CpG-2007 has better protection against *Edwardsiella tarda* infection than CpG-1668 (112). In grass carp, CpG-1670A, which contains three copies of the AACGTT-hexamer motif in 25 nucleotides, displays a greater capacity to protect teleosts against viral infection than CpG-1668 and CpG-2006 (116).

The ability of teleost TLR9 and TLR21 to uniquely distinguish different types of CpG-hexamer motifs in teleosts may account for the different CpG-ODN sequences that have been reported to participate in the induction of immune responses in different teleost species. The zebTLR9 has been shown to broadly recognize different CpG-hexamer motifs; however, it more strongly recognizes CpG-ODN with the GACGTT- or AACGTT-hexamer motif than CpG-ODN with the GTCGTT-hexamer motif. In contrast, zebTLR21 responds more to CpG-ODN with the GTCGTT-hexamer motif. Further study suggests that CpG-ODNs with an optimized sequence for activating these two TLRs can generate the strongest immunostimulatory activity in this species (86). CpG-ODNs with the GTCGTT-hexamer motif, such as the CpG-2722 and CpG-2727, have strong effects on the TLR21 group like that required for the activation of zebTLR21; in contrast, CpG-1826 with the GACGTT-hexamer motif does not activate this TLR (99). CpG-2006, CpG-2007, and CpG-1826 are reportedly able to activate TLR21 in large yellow croakers (109). The optimized sequence for CpG-ODN to strongly activateTLR9s or TLR21s from other teleosts has not been investigated. Given the large diversity in teleost species, there is not expected to be a universal CpG-ODN sequence for strong activation of TLR9 or TLR21 from different teleost species. This means that the interaction of CpG-ODN with TLR9 or TLR21 from different teleost species must be investigated individually to generate conclusions about how to design a sequence for CpG-ODN with a strong immunostimulatory activity in the teleost species.

## Functional Activity of Teleost TLR9 and TLR21 IN Response to CPG-ODN Stimulation: Suggestions Made by Their Structure

Along with the requirement of an optimized nucleotide sequence for CpG-ODN to strongly activate TLR9 and TLR21, whether CpG-ODN can generate a strong immune response in a teleost species is also determined by the intrinsic functional activity of TLR9 and TLR21 in that teleost. Furthermore, although both TLR9 and TLR21 in zebrafish are active in response to CpG-ODN stimulation (86), it is still unclear whether both are functional in other teleost species. Nevertheless, some suggestions can be made from the protein sequences analysis of these two TLRs from different teleost species and the study of the structure/functional activity relationship of mammalian TLR8.

**Figure 1:**
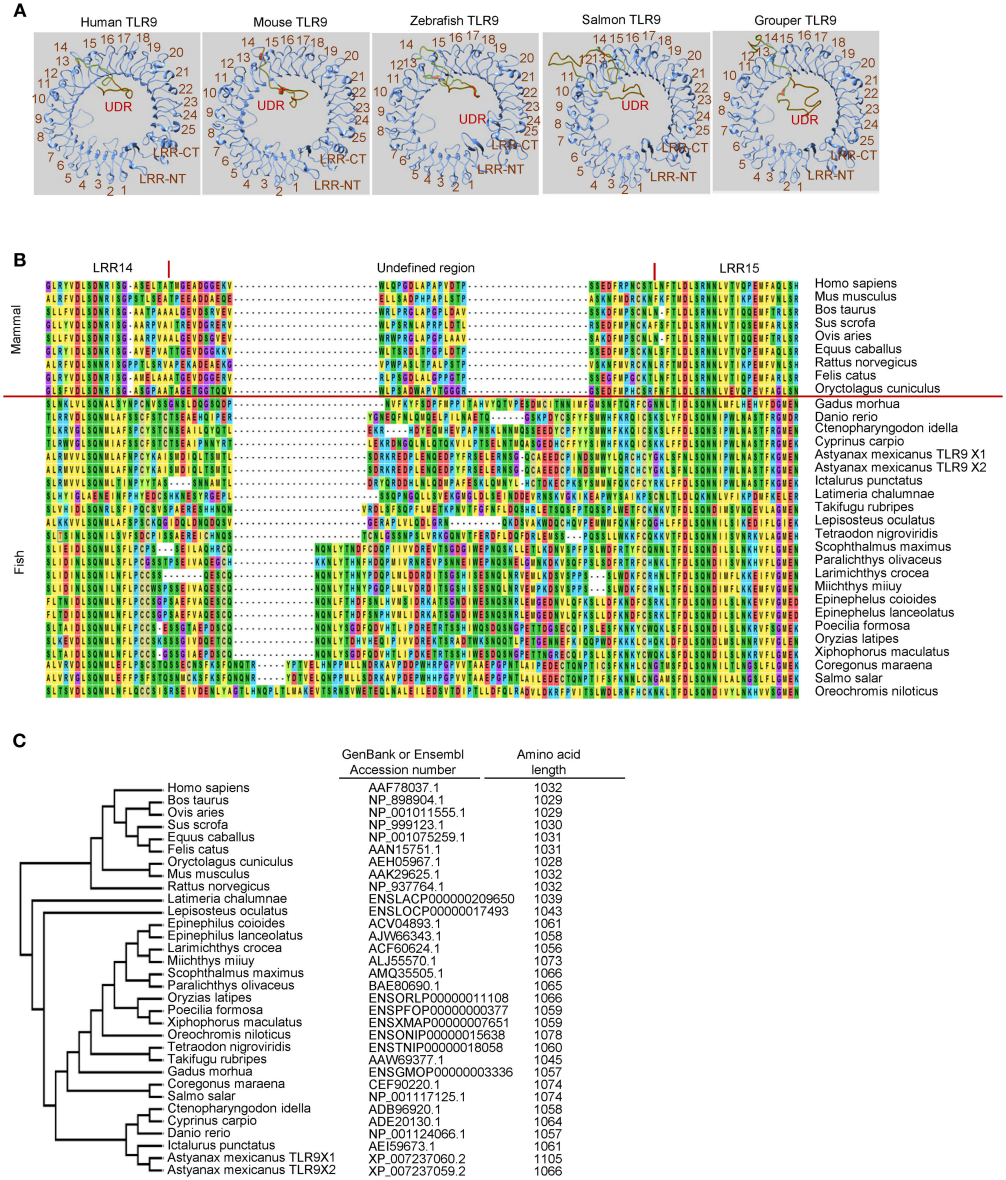
Undefined region of toll-like receptor 9 (TLR9) from different teleost species. **(A)** Computational modeling of the ectodomain protein structures of TLR9 from different species as indicated. These structural models were predicted with SWISS MODEL (www.swissmodel.expasy.org). **(B)** Alignment of protein sequences for the undefined regions between leucine-reach repeat (LRR)14 and LRR15 in the ectodomain of TLR9 from different species. Multiple alignments of the amino acid sequences of TLR21s were performed using ClustalW2 (www.ebi.ac.uk/Tools/msa/clustalw2). **(C)** Phylogenetic analysis of TLR9s from different species. The GenBank accession numbers of these TLR9 protein sequences are listed in the left column. Numbers in the right column are the amino acid lengths of these TLR9s.

In mammals, TLR7, TLR8, and TLR9 are phylogenetically closely related and are a subfamily of TLRs (5, 8). These three TLRs have an ectodomain in a horseshoe-like shape that consists of 25 copies of LRRs and a unique undefined region (also called a Z-loop) between LRR14 and LRR15 (122, 123), as shown in Figures 1A,B for the ectodomains of TLR9s. This unique undefined region plays an important role in ligand activation of members of this TLR subfamily (124–126). Previous studies have shown that TLR8s from several non-rodent species, including cat, horse, sheep, and bovine, are activated by their agonists; whereas, TLR8s from the mouse and rat, two rodent species, do not respond to ligand stimulation (127). Another study revealed that rabbit TLR8 (also a rodent TLR8) has very little activity after ligand stimulation compared to that of humans (128). Inspection of the ectodomains of these TLR8s reveals that the lengths of amino acid residues within the undefined regions varies between TLR8s from the non-rodent group and those from the rodent group. Compared to non-rodent TLR8s, the undefined regions of mouse and rat TLR8s are shorter by five amino acid residues; whereas, the undefined region of rabbit TLR8 is longer by 34 amino acid residues. Although the structural base is still unclear, it has been suggested that the lesser functional activity of these rodent TLR8s is a result of the varied lengths of their undefined regions (127, 128). Distinct from TLR8, non-functional TLR7 and TLR9 have not been reported in mammalian species. Consistently, the length of the undefined regions in mammalian TLR7s and TLR9s are more conserved than that in the TLR8s (127, 128).

Like mammalian TLR9s, teleost TLR9s also contain an undefined region in their ectodomain, which results in an extruded loop in the horseshoe-shaped ectodomain of these TLRs (Figures 1A,B). Interestingly, there are large variations in the length of undefined regions in teleost TLR9s. The regions in teleost TLR9s are longer than in mammalian TLR9s (Figure 1B). Moreover, the length of these undefined regions is more consistent in the more phylogenetically-related teleost TLR9s than in the more distantly-related teleost TLR9s. For example, TLR9s of zebrafish, grass carp, common carp (*Cyprinus carpio*), Mexican tetra (*Astyanax mexicanus*), and channel catfish (*Ictalurus punctatus*) are more closely phylogenetically related, and the lengths of their undefined regions are more consistent than in the TLR9s of the Atlantic salmon and orange-spotted grouper, which are more distantly related (Figures 1B,C). Given that the undefined regions play a role in the functional activity of TLR8, this structural analysis of undefined regions within TLR9s from different teleosts suggests that there is a large difference in the intrinsic functional activities of TLR9s from different teleost species. Furthermore, because the more phylogenetically-related teleost TLR9s contain more conserved undefined regions, it also suggests that there are more similar functional activities for the more closely related teleost TLR9s.

**Figure 2:**
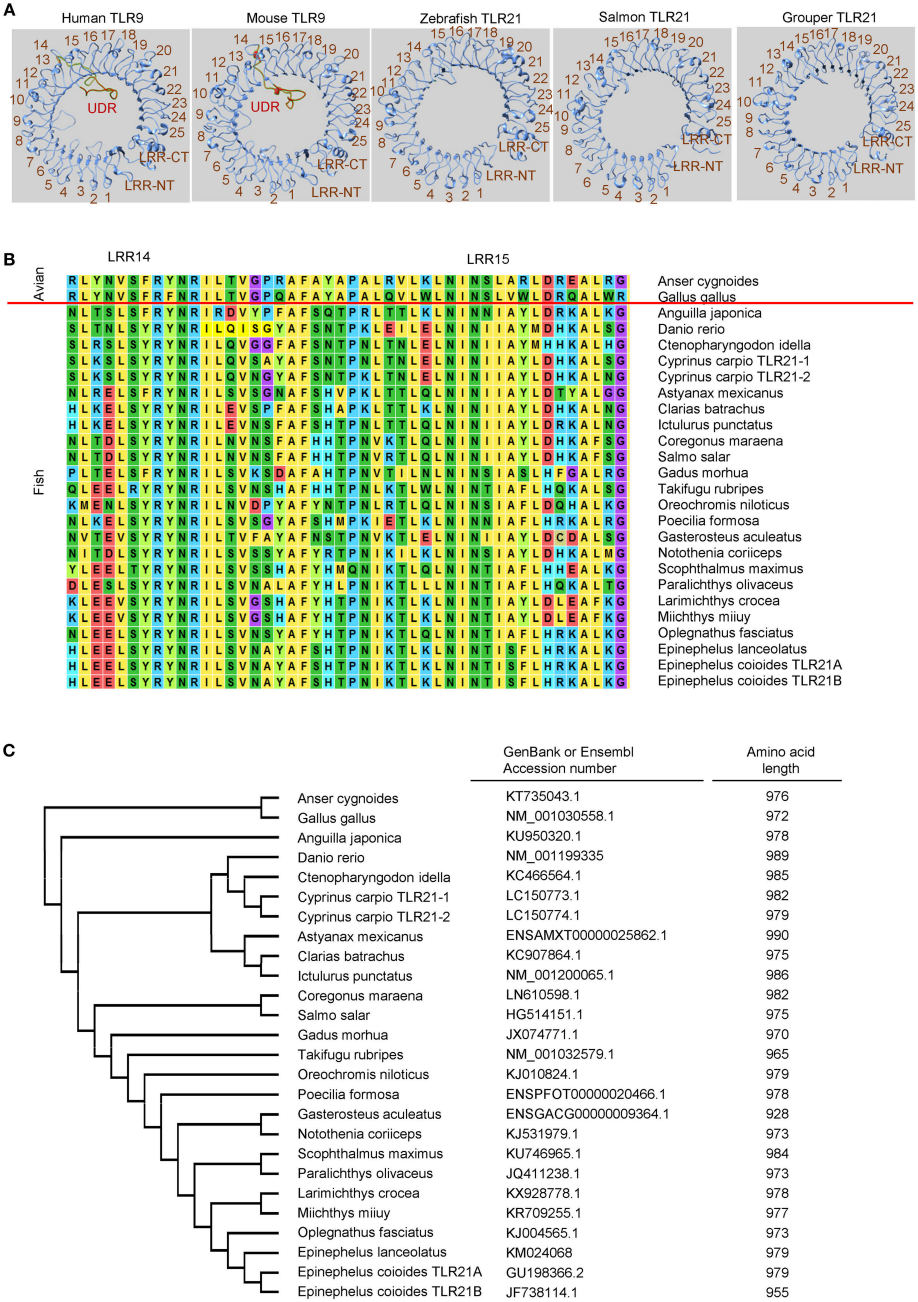
Toll-like receptor 21 (TLR21) from different teleost species does not contain an undefined region. **(A)** Computational modeling of the ectodomain protein structures of TLR21 from different species as indicated. These structural models were predicted with SWISS MODEL (www.swissmodel.expasy.org). **(B)** Alignment of protein sequences for the regions from leucine-reach repeat (LRR)14 to LRR15 in the ectodomain of TLR21 from different species. ClustalW2 (www.ebi.ac.uk/Tools/msa/clustalw2) was used to perform multiple alignments of the amino acid sequences of TLR21s. **(C)** Phylogenetic analysis of TLR21 from different species. The GenBank accession numbers of these TLR21 protein sequences are listed in the left column. Numbers in the right column are the amino acid lengths of these TLR21s.

In contrast, although TLR21 is functionally related to TLR9 in response to CpG-ODN stimulation, TLR21 is more phylogenetically related to members of the TLR11 subfamily and is an ortholog closer to the TLR13 subfamily (129, 130). Analysis of chicken TLR21 revealed that it does not have an undefined region, as in TLR9. In addition, a study of TLR21 proteins from different species shows that these TLR21s are highly homologous (130). The same is true for teleost TLR21s. As Figure 2 illustrates, undefined regions are not found in teleost TLR21s whether the TLR21s are closely related or distantly related to each other; therefore, the highly-diversified ectodomains of TLR9s from different teleost are not observed in teleost TLR21s. In general, the teleost TLR9s contain more than 1,000 amino acid residues, and the teleost TLR21s have < 1,000. A lack of the undefined region in these teleost TLR21s is the main reason why teleost TLR9s contain more amino acid residues than TLR21s (Figures 1C, 2C). The more conserved ectodomains of teleost TLR21s suggest a more stable functional activity of TLR21s within different teleost species. Nevertheless, these suggestions made by structural analyses of the teleost TLR9s and TLR21s are waiting for confirmation by experimental investigation.

## Expression and Tissue Distribution of TLR9 and TLR21 in Teleosts

In addition to their functional activity, the differential expression levels and tissue distributions of TLR9 and TLR21 are likely to be another level of determinant of CpG-ODN efficacy in teleosts. The expression profile of TLR9 has been investigated in several different species of teleost and has been shown to be broadly expressed in different tissue types and development stages (131–135). In gilthead sea bream (*Sparus aurata*), the expression levels of TLR9 transcripts are detected in the gill, head kidney, and spleen (119). In channel catfish, TLR9 is expressed in the skin, gill, head kidney, and spleen (135). In addition, TLR9 expression is inducible by responding to different stimuli and microbial infections (105, 135). For example, TLR9 is broadly expressed in larval, juvenile, and adult stages of cobia (*Rachycentron canadum*) in all analyzed tissues, including the gill, intestine, head kidney, liver, skin, and spleen. Cobia challenged with *Photobacterium damselae* subsp. *piscicida* results in increased TLR9 expression in these tissues with different dynamic profiles (105). TLR9 expression in the skin and gills of channel catfish is induced by infection with *Ichthyophthirius multifiliis* (135).

TLR21 has an expression profile like that of TLR9. In yellow catfish, the TLR21 gene is detected in fertilized eggs and in the young up to 30 days after hatching. In adult fish, this gene is detected in the muscles, stomach, skin, swim bladder, midgut, brain, spleen, trunk kidney, skin mucus, head kidney, liver, heart, gill, and blood, with the highest expression in the spleen. TLR21 mRNA expression levels in the spleen, head kidney, trunk kidney, liver, and blood of yellow catfish are upregulated after challenging the fish with killed *Aeromonas hydrophila* (136). In turbot (*Scophthalmus maximus*), TLR21 transcripts are broadly expressed in different tissues, with the highest expression in the spleen followed by the head kidney and liver. In addition, after infection with turbot reddish body iridovirus or stimulation with polyinosinic:polycytidylic acid and CpG-2395, which contain a GTCGTT-hexamer motif within 22 nucleotides, the expression of the turbot TLR21 transcript is upregulated in the gills, head kidney, spleen, and muscle (114). In large yellow croakers, TLR21 is expressed in all tested tissues, with higher levels in immune-related tissues such as the spleen, head kidney, and gills (109). In rock bream, TLR21 transcripts are ubiquitously expressed in different tissues, with higher expression in the spleen followed by the liver and blood. In contrast, the kidney, heart, gill, head kidney, and skin have lower expression levels of these transcripts. In addition, mRNA of the rock bream TLR21 is significantly upregulated in the spleen after stimulation with *Streptococcus iniae*, rock bream iridovirus, and *Edwardsiella tarda* (137).

Interestingly, the induction of gene expression in different tissues of cobia by CpG-ODNs is reported to be CpG-ODN-sequence dependent. CpG-1668 and CpG-2006 induce high expression levels of TLR9 in the spleen; whereas, CpG-1668 is more potent in the induction of TLR9 expression in the liver. In the liver and spleen, CpG-1668 and CpG-2006 induce higher expressions of IL-1β and CC chemokines than CpG-2395 and the control CpG-2137; however, in these tissues, CpG-2006 induces high levels of immunoglobulin M (IgM), and CpG-2395 induces high expression levels of Mx (105). The underlying reason for this CpG-ODN sequence- and tissue type-dependent induction of gene expressions is unclear. However, it may reflect that the different expression levels of TLR9 and TLR21 in a tissue type and the ability of TLR9 and TLR21 to differentially recognize different type of CpG-ODN are the main causes for the different activity levels of a CpG-ODN in different tissues.

## Conclusion and Perspectives

Most of the knowledge on how to design a nucleotide sequence for CpG-ODN to achieve strong *in vivo* immunostimulatory activity has come from early studies on mammalian species that express TLR9 only and not TLR21. The discovery of TLR21 as another CpG-ODN receptor in teleosts may explain why previous experience on the activities of various CpG-ODNs in mammals cannot be replicated in teleosts. This also suggests that more understanding of both TLR9 and TLR21 is required for design of CpG-ODN sequence to have strong activity in teleosts. Given that the functional activity of TLR9 and TLR21 may vary among different teleosts, further investigations with cell-based TLR9 and TLR21 activation assays are required to determine whether both TLRs in a teleost are functional or if only one of the two TLRs has the dominant functional activity.

Aquaculture is one of the fastest growing areas of agriculture. The production of farmed teleosts has exceeded that of captured teleosts. Farmed teleosts are susceptible to viral, bacterial, and parasitic infections. Thus, effective immune modulators, including vaccines, and vaccine adjuvants, are required for the aquaculture of farmed teleosts (138–140). CpG-ODN has proven to be an effective adjuvant and antimicrobial agent in teleost (53, 55). The approval of its usage as a vaccine adjuvant in humans (51, 52) further supports its effectiveness and safety as an immunostimulant in agricultural areas, including aquaculture, for food production.

## Author Contributions

C-YL, G-YY, YL, RX, and T-HC the review results from the opinions and concepts of all authors listed. The review was written by C-YL and T-HC with the help of G-YY, YL, and RX.

### Conflict of Interest Statement

The authors declare that the research was conducted in the absence of any commercial or financial relationships that could be construed as a potential conflict of interest.
